# Home-grown school feeding: promoting local production systems diversification through nutrition sensitive agriculture

**DOI:** 10.1007/s12571-017-0760-5

**Published:** 2018-01-29

**Authors:** Samrat Singh, Meenakshi Fernandes

**Affiliations:** 1 Partnership for Child Development, School of Public Health, Imperial College London, Norfolk Place, London W2 1NY, UK

**Keywords:** Production diversity, Dietary diversity, Sustainable food systems, Policy approaches, Structured demand, Sub-Saharan Africa

## Abstract

The consumption of some non-staple crops such as legumes and dark, green leafy vegetables can address common deficiencies in key nutrients such as vitamin A and iron; however, limited markets and supply chain development impede their production and accessibility to consumers. This study investigates the pathways to promote agricultural production and dietary diversity for a local market intervention called Home-Grown School Feeding (HGSF). School feeding menus from 24 districts across 10 regions in Ghana during the 2014–15 school year were analysed in terms of food groups and several individual foods. The menus were then compared with food groups produced by households during the past year or consumed in the past seven days using data collected from a household survey. Greater inter-food group diversity in the menus was associated with higher production levels for tubers and dark, leafy green vegetables in the South and cereals in the North. A correspondence between the frequency in which a food group appeared in a menu and the share of households who consumed foods from the food group was also noted. Key issues, such as optimizing supply chains, enabling farm linkages and supporting diverse nutrient rich food groups, that underlie the success of Home-Grown School Feeding and other agricultural policies with similar goals of promoting production and dietary diversity are highlighted through commodity specific examples. The findings of this study may help strengthen operational linkages between agriculture production and nutrition for HGSF and other similar interventions.


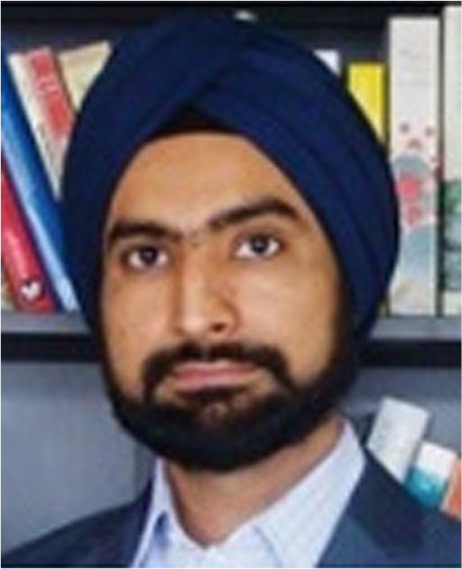



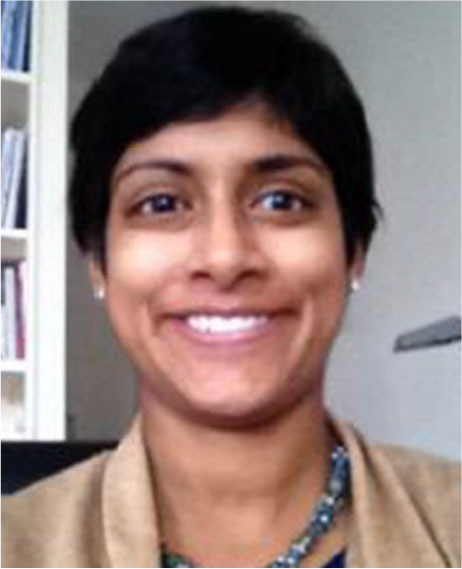


## 1 Introduction

Evidence suggests that the mono-culturization of agriculture production towards staple foods-based systems in developing countries has crowded out the production of other food groups such as legumes and vegetables (Kataki 2002; Pingali 2015). This may have serious implications for the availability and consumption of non-staple food groups and thereby common deficiencies in nutrients such as protein, iron and vitamin A (Negin et al. 2009; DeClerck et al. 2011). Policies that promote diversity of food supply may help to rectify the imbalance in such food systems (Hawkes 2007; Remans et al. 2014; Pingali 2015).

A growing literature highlights the pathways among agricultural production, dietary diversity and nutritional outcomes which are multiple and context-specific (Jones et al. 2014; Fanzo et al. 2013). Gender is one critical pathway as women exhibit a high level of participation in the production and marketing of some non-staple, nutrient-rich crops, including neglected and underutilised species (Mayes et al. 2011; Malapit and Quisumbing 2015). Women are also more likely to undertake regular management activities such as weeding, cleaning and grading which are more common for vegetables (Joshi et al. 2006). Other studies have found that smallholder farmers are more likely to be engaged in production and marketing of non-staple foods including vegetables and legumes (Joshi et al. 2006; IFAD 2014). However, significant challenges adversely affect market conditions and integration for non-staple food production (Pingali et al. 2005; Barett 2008). These include high transaction costs linking production with value chains, limited incentives for farmers in terms of prices (Joshi et al. 2003), and the perishable nature of some non-staple food groups such as fruits and vegetables (FAO 1981; McKee 2012).

Realigning macro-level agricultural policies, such as those linked to output and input support, is important to create a more balanced incentive structure. However, this alone is not sufficient. Over time, the bias favouring the production of staple crops has, as cause and effect, deeply influenced household level production and consumption behaviour with implications for diets and nutrition (Ecker and Quaim 2011). Diversifying production to non-staple foods would require a systematic and sustained localized approach to incrementally build viable market conditions. In community-based peasant farming which is predominant in most parts of sub-Saharan Africa, market systems are primarily atomistic based around a few communities. Thus, conventional broad based market support instruments which are premised on well-integrated markets, and incentive responses designed around staples are likely to have limited impact. Localized market interventions can potentially address some of these challenges through strengthened commodity-specific value chains. One such intervention is ‘Home Grown School Feeding’ (HGSF) through which locally-sourced meals are provided daily to children attending schools. In 2014, at least 47 countries in sub-Saharan Africa were implementing school feeding programmes, of which at least 20 were HGSF or similar models.

Key principles of HGSF include local food procurement, smallholder engagement, nutrient-rich and diverse foods, and regularity in meal provision (Gelli et al. 2010). When appropriately designed, school meal menus can help ensure an effective application of these principles. Several studies have illustrated the potential impact of HGSF to create demand for cereals and other staple foods, leading to increased farmer incomes and improved livelihoods (Masset and Gelli 2013; Sumberg and Sabates-Wheeler 2011; Gelli et al. 2010). However, the potential for HGSF to support diversified food systems has been less explored.

This study investigates how HGSF may support diversified food systems in the context of Ghana. The Ghana School Feeding Programme (GSFP) provided daily meals to an estimated 1.7 million children, or about one out of every three primary school children in 216 districts of the country during the 2014–15 school year (GSFP Secretariat 2015). Meals were based on weekly menus that districts develop each school year and that may be adapted to the local context in terms of agricultural production, food culture and preferences (Parish and Gelli 2015). Specifically, this study presents a framework, relating school meals to local production and consumption patterns, and applies it to a set of 24 districts located across Ghana. The investigation considers food groups as well as some specific commodities to illustrate how HGSF may support diversified food systems through different pathways including women’s empowerment, market integration and supply chain management.

## 2 Materials and methods

The analysis draws from two sources of data. The first source was household survey data collected from the same 24 districts that included information on household dietary consumption and agricultural production. These data were collected as part of a baseline survey for an impact evaluation of the GSFP (Gelli et al. 2016). The impact evaluation sought to assess the impact of the programme on a wide range of child and community outcomes including education, nutrition and agriculture (Gelli et al. 2016). In total, the evaluation focused on a set of 60 districts in Ghana out of a total of 216 districts. HGSF was introduced in 30 of these districts while the other 30 districts served as a control group. Two comparable public primary schools and the surrounding communities were selected from each district.^1^ Household listings were compiled in each enumeration area (EA) by the survey team supervisors assisted by community leaders. The list of all households with a child aged five to 17 years of age in each EA constituted the sampling frame from which participating households were selected at random for the household questionnaire. About 20 to 25 households were selected from each community for the survey. The questionnaires were administered by teams of enumerators from the University of Ghana in the local language. The responses were input and cleaned at the University of Ghana.

The second source of data was a set of school feeding menus from 24 districts from the HGSF arm of the impact evaluation. Menus were developed using a tool known as the School Meals Planner (Partnership for Child Development 2014; Fernandes et al. 2016). The tool supports the design of menus that link dishes with the types and quantities (in grams) of each individual food. Menus were used in district schools that participated in the Ghana School Feeding Programme (GSFP) during the 2014–15 school year. But menus for the remaining districts in the home-grown school feeding arm of the impact evaluation could not be obtained for several reasons. For example, the school sampled for the evaluation may not have offered school feeding or a menu was not developed for the district with the School Meals Planner tool. In the HGSF context ‘local’ can be defined in different ways depending on the context (Sumberg and Sabates-Wheeler 2011). For the analysis presented in this study, ‘local’ is defined as the district according to governance structure in Ghana. For each menu, the number of individual foods from each food group, as well as the quantity of each food group in grams was calculated. Inter-food group diversity was defined as the number of food groups. Intra-food group diversity was defined as the number of unique individual foods in a food group.

**Fig. 1:**
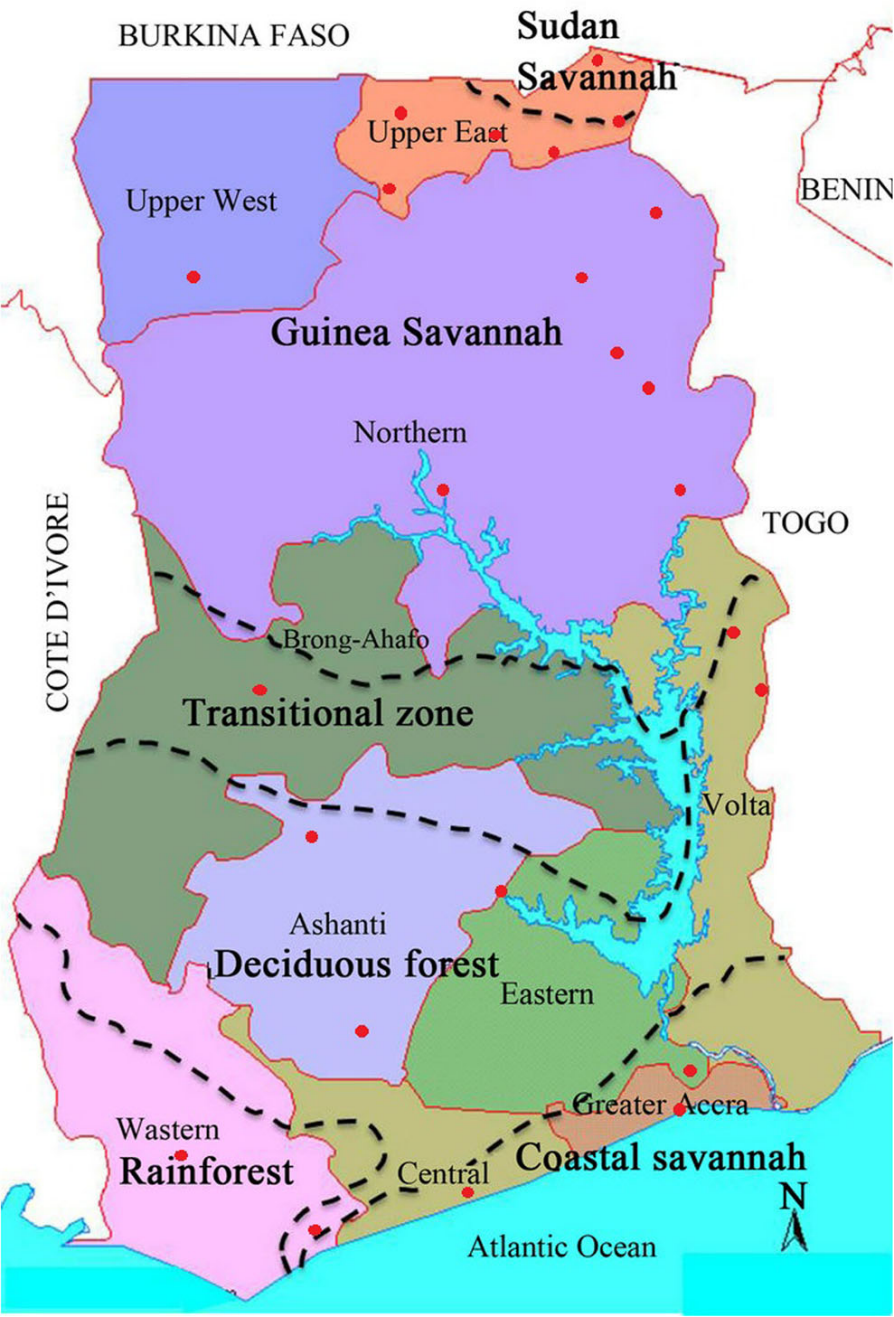
Distribution of agro-ecological zones and location of the 24 study districts. Notes: Agro-ecological zones are delineated by the dotted lines and include: Rainforest, Coastal savannah, Deciduous forest, Transitional zone, Guinea savannah, and Savannah. The red dots indicate the districts represented in the study. The map is based on a figure from Kemausuor et al. 2013

Figure 1 illustrates the distribution of the 24 study districts across the ten regions of Ghana. The country encompasses six distinct agro-ecological zones in addition to more than 100 ethnic groups, reflecting diverse food cultures and agricultural production practices. Northern Ghana, which includes the three regions of Upper West, Northern and Upper East in the north, is typically distinguished from Southern Ghana, which includes the remaining seven regions of the country. Poverty and food insecurity are concentrated in Northern Ghana, while the national capital Accra is located in Southern Ghana (GSS 2012). Thirteen of the 24 study districts were located in Northern Ghana.

### 2.1 GSFP menus

Individual foods in the menus were classified into the following five food groups: 1) cereals; 2) tubers; 3) legumes (including nuts and seeds); 4) dark, green leafy vegetables and 5) other vegetables (Kennedy et al. 2013). Dark, green leafy vegetables, including individual foods such as cassava and cocoyam leaves, were defined as a separate food group apart from vegetables, given their nutritional value, in particular non-haem iron and vitamin A, as well as their prominence in the Ghanaian diet. Fruits were not analysed due to their minimal inclusion in the menus. Common cereals included rice and maize, while cassava and cocoyam were tubers that featured prominently. Legumes that appeared frequently in the menus included cowpeas,^2^ groundnut^3^ and melon seeds, while tomatoes and okra were common ‘other vegetables’.

### 2.2 Household survey data

Survey data were collected from a sample of households in the same 24 districts between June and August 2013 (Gelli et al. 2016). The data included a total of 1009 households who reported their dietary consumption in the past seven days. Of these, 621 were farming households who reported undertaking any agricultural production in the past year. Survey enumerators asked households to specify which individual foods they produced and consumed using a predefined list. There were differences among the individual foods included in the production and consumption list. The agricultural production list included 42 individual foods as compared to 59 individual foods for household food consumption. The responses were classified into the same five food groups defined for the menu analysis in Section 2.1. Households could also provide open responses for both the production and consumption questions. These responses were reviewed and classified into the five food groups when applicable. In most cases the individual foods were classified into one food group. However, cassava and cocoyam were included in both the tubers and dark, green leafy vegetables food groups even though they may have only been cultivated for the tuber.

## 3 Calculation

A descriptive analysis by food groups and the selected individual food items was then undertaken for the menu and household survey data from the set of districts in Ghana. The share of households in the district that were producing or consuming foods from each food groups was calculated. Separate indicators were constructed for four individual, non-staple foods that were commonly featured in the menus and that were also included in both the agricultural production and diet modules of the household survey. These individual food items were groundnut and cowpeas (classified in the legumes food group), okra and tomatoes (both classified in the other vegetables food group). The statistical significance of differences in production and consumption between Northern and Southern Ghana were assessed for analyses using the household survey data, but not the menu data due to the limited sample size.

In addition, a simulation was undertaken to estimate the overall potential national market demand from the GSFP for each of the five food groups based on information from the 24 district menus. The simulation assumed that all schools participating in the GSFP followed a menu from one of the 24 study districts. GSFP schools located in regions that included only one of the study districts were assumed to follow the menu from that study district. In other regions with multiple study districts, the minimum and maximum per-child quantity of each food group from the set of menus provided the basis for lower and upper bound estimates used, respectively. These estimates for the weekly quantities of each food group per child were multiplied by the number of children enrolled in schools participating in the GSFP in that region, and the number of school weeks in the 2014–15 school year, which was provided by the head office for the programme (GFSP Secretariat 2015).

## 4 Results

### 4.1 Menu analysis

The menus included the names of dishes prepared each school day (Monday through Friday) for children attending the school as well as the individual foods and quantities needed for each dish. For example, in one district, children may receive bean stew and plain rice on Monday. The menu would list this dish as well as each of the individual foods in the dish such as beans, rice, onion and tomatoes as well as the quantity in grams per child. Caterers were instructed to purchase sufficient amounts of the individual foods every week based on the quantities provided in the menu and the number of children attending the school. In addition to purchasing the individual foods, the caterers prepared the meals every day for the school children. The meals were prepared on the school premises or brought to the school from another locale.

**Fig. 2:**
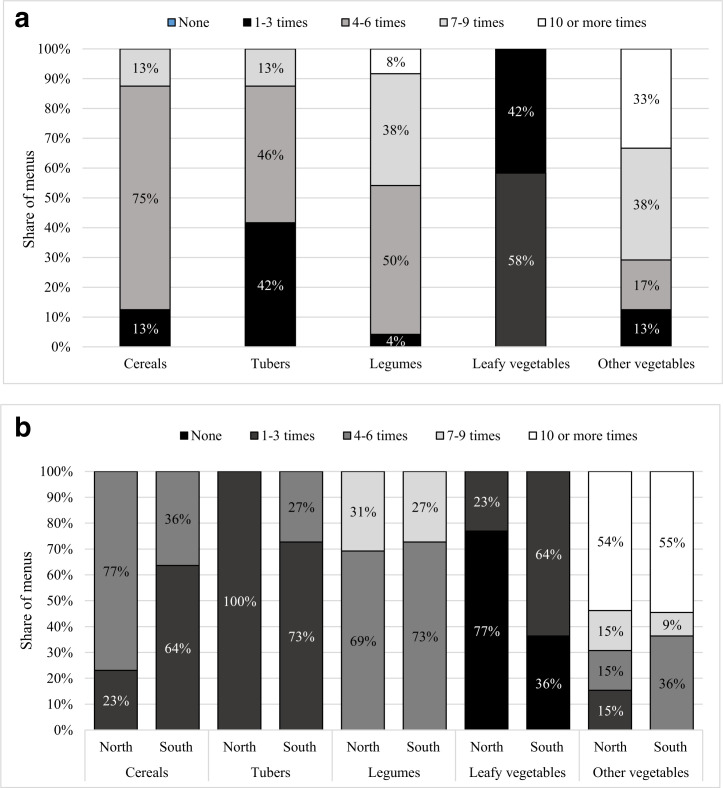
**a** Intra-food diversity in menus, overall. Notes: Sample was 24 district menus from Ghana. The definition of food groups follows Kennedy et al. 2013. **b** Inter-food diversity in menus, by geographic region. Notes: Sample was 24 district menus from Ghana. The definition of food groups follows Kennedy et al. 2013

All 24 menus included all five of the food groups defined by the study. Figure 2a presents the intra-food group diversity of the menus overall, while differences between Northern and Southern Ghana are presented in Fig. 2b. The greatest intragroup diversity was observed for other vegetables. Eight menus had four to six individual foods from this group. Legumes had the next highest intra-group diversity. Two menus had four to six individual foods from this group. Most menus (88%) included two cereals, and one or two tubers. More than half of menus (58%) did not include dark, leafy green vegetables. More individual foods from the cereals and legumes food groups were included in menus from Northern Ghana, while more individual foods from the tubers, dark, leafy green vegetables and other vegetable food categories were included in menus from Southern Ghana.

### 4.2 Household survey analysis

Table 1 presents findings from the household survey analysis regarding agricultural production. Cereals were the most commonly grown with 88% of farming households producing at least one individual food in the food group. Other vegetables were the least commonly reported (10%). Cereals and legumes were more likely to be produced in Northern Ghana (p < 0.001). Tubers and dark, green leafy vegetables were more commonly grown in Southern Ghana (p <0.001).

**Table 1:** Share of households producing individual foods in the five food groups during the past year, by geographic region

Food group:	Overall (*n* = 630)		North^a^ (*n* = 452)		South^b^ (*n* = 178)	
	Mean	SD	Mean	SD	Mean	SD
Cereals	0.88	0.32	0.95	0.21	0.70	0.46 ***
Tubers	0.41	0.49	0.26	0.44	0.77	0.42 ***
Legumes	0.49	0.50	0.64	0.48	0.12	0.33 ***
Dark, green leafy vegetables	0.32	0.47	0.17	0.38	0.71	0.46 ***
Other vegetables	0.10	0.30	0.10	0.30	0.10	0.30

Sample limited to farming households in the 24 study districts of Ghana. Survey data collected in Summer 2013. Food groups defined as per Kennedy (2013). SD = standard deviation. T-tests used to assess North/South differences: * *p* <0.05, **, <*p* <0.01, *** *p* <0.001

a Includes Northern, Upper East and Upper West regions

b All other seven regions of Ghana

Table 2 presents findings for household food consumption as reported in the survey. Almost all households reported consumption of cereals in the past seven days (99%), and all households reported consumption of other vegetables. Consumption of legumes and tubers were lower than consumption of cereals (80 and 78% respectively), but still high relative to the consumption of dark, leafy green vegetables (37%). Differences by geographic region mirrored those found for agricultural production. Legume consumption was more common in the North as compared with the South (93 versus 64%, p < 0.001). Consumption of tubers and dark, leafy green vegetables were more prevalent in the South as compared with the North (95 and 70% versus 64 and 11% respectively, p <0.001).

**Table 2:** Share of households consuming individual foods in the five food groups during past seven days, by geographic region

Food groups:	Overall (*n* = 1055)		North^a^ (*n* = 584)		South^b^ (*n* = 471)	
	Mean	SD	Mean	SD	Mean	SD
Cereals	0.99	0.09	1.00	0.04	0.98	0.13 **
Tubers	0.78	0.42	0.64	0.48	0.95	0.21 ***
Legumes	0.80	0.40	0.93	0.25	0.64	0.48 ***
Dark green, leafy vegetables	0.37	0.48	0.11	0.31	0.70	0.46 ***
Other vegetables	1.00	0.05	1.00	N/A	0.99	0.08

Sample limited to farming and non-farming households in the 24 study districts of Ghana. Survey data collected in Summer 2013. Food groups defined as per Kennedy (2013). SD = standard deviation. T-tests used to assess North/South differences: *p <0.05, **,*p*<0.01, ***p<0.001

a Includes Northern, Upper East and Upper West regions

b All other seven regions of Ghana

### 4.3 Individual food analysis using menus and survey data

The inclusion of certain food groups and individual foods in menus may strengthen their value chains and reduce post-harvest losses, particularly those that are more commonly cultivated by smallholder farmers and women. This section presents findings from the individual food analysis for groundnut and cowpeas (classified in the legumes food group), okra and tomatoes (both classified in the other vegetables food group). Groundnut is a major source of protein in the Ghanaian diet and production is mainly undertaken by smallholder farmers with less than two hectares of arable land (Angelucci and Bazzucchi 2013). Cowpea is another important legume in Ghana. Most of the production and marketing activities for this crop in sub-Saharan Africa are undertaken by women farmers (FAO 2004). In a pilot study from Northern Ghana, at least 40% of additional income from improved cowpea production in Ghana accrued directly to women farmers (ICRISAT/IITA/CIAT 2012). Okra is traditionally a rainy season crop that is cultivated by women and may be grown in mixed cropping with other vegetables (Kumar et al. 2010). The production of tomatoes is characterised by weak market access along the value chain and scattered small farm production (Esther 2015).

**Fig. 3:**
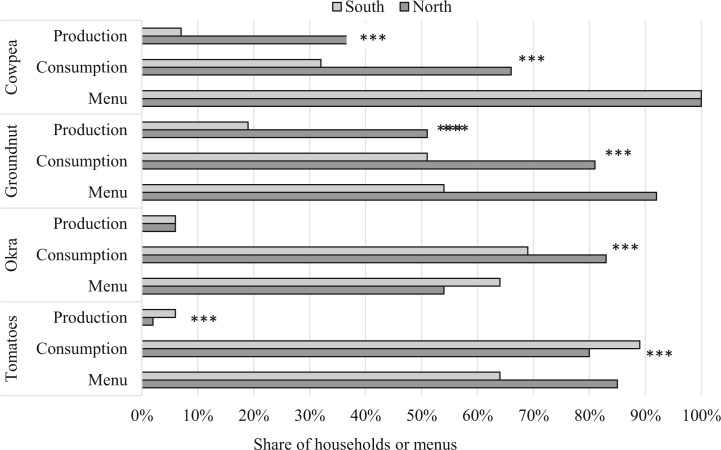
Share of households producing and consuming individual foods, and inclusion of individual foods in menus, by region. Notes: Sample limited to farming and non-farming households in the 24 study districts of Ghana. North includes Northern, Upper East and Upper West regions while South includes the other seven regions of Ghana. Survey data collected in Summer 2013. Food groups defined as per Kennedy (2013). T-tests used to assess North/South differences in survey data results: * *p* < 0.05, **, *p* < 0.01, *** *p* <0.001

Figure 3 presents results from the menu analysis and survey data for these four individual foods. All the menus included cowpeas, while okra, fresh tomatoes and groundnut were commonly featured. Large regional differences were noted in several instances. Groundnut was more common in menus from the North (90% versus 50% in the South). Production and consumption of both groundnut and cowpeas were higher in the North (*P* < 0.001), which is consistent with findings for the overall legume food group results presented in Section 4.2. In total 20 out of the 24 menus featured okra and tomatoes, which were commonly consumed by households. The share of households producing these crops, however, was low. Although not included in the analysis, 88% of menus included processed tomatoes which were in tinned or paste form.

### 4.4 HGSF market demand for diverse crops

**Fig. 4:**
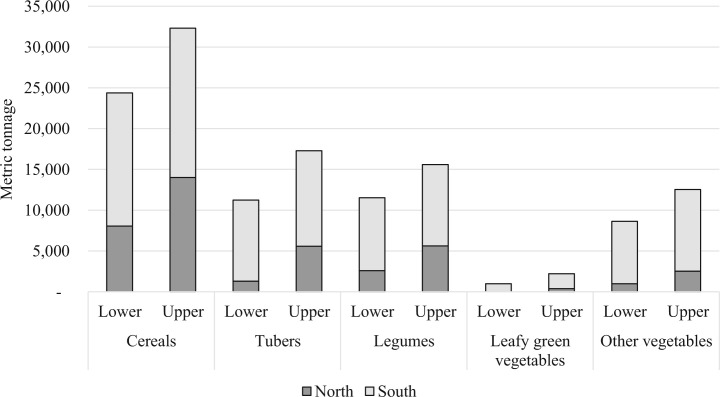
Simulation results for annual, national demand from the GSFP for the five food groups. Notes: a) North includes Northern, Upper East and Upper West regions while South includes the other seven regions of Ghana. Food groups defined b) The lower bound was extrapolated based on the menu from the region with the lowest quantity of the food group. The upper bound was extrapolated based on the menu from the region with the highest quantity of the food group

Figure 4 presents the estimated market demand of the GSFP for the five food groups during the 2014–15 school year. The decomposition by region reflects the distribution of enrolment in the GSFP across the country. About 32% of children eligible for GSFP meals in the school year were in Northern Ghana. The greatest quantities were generated for the cereals food group (24,376 to 32,306 metric tonnes). The quantities generated for legumes (11,532 to 15,588 metric tonnes) and tubers (11,235 to 17,279 metric tonnes) were each about half of the amount of cereals. The demand for other vegetables was also sizeable (8641 to 12,531 metric tonnes). The simulated demand for dark, leafy green vegetables and other vegetables was higher in Southern Ghana, reflecting the greater prominence of these food groups in the menus in the South.

## 5 Discussion

Institutionalized procurement that is decentralized to the local level through programmes such as HGSF may induce general and commodity-specific corrective measures to promote diversified food production and consumption and may specifically benefit smallholder farmers and women (Banerjee 2011). For HGSF, school menus provide a critical interface to strengthen linkages among agricultural production, markets and diets (Fernandes et al., forthcoming). It is important to bear in mind that for HGSF to effectively induce these changes in the market, the programme must include specific mechanisms to enable smallholder farmer participation and linkages to input support services. Given that smallholder farmers are highly risk averse, the structured demand needs to be demonstrably stable over time to catalyse market participation. It is therefore critical that whilst fulfilling basic nutrition parameters, menu design in selecting food should address agro-ecologically suitability and financial viability.

This study identifies and explores the pathways through which HGSF menus may provide an interface to promote agricultural production diversity and dietary diversity in Ghana. All menus included four or all five of the studied food groups, while intra-food group diversity was high for legumes and other vegetables. More inter-food group diversity for tubers and dark, leafy green vegetables in the South was reflected in a higher share of households producing these foods in this region as compared to the North. Conversely, the greater diversity of cereals in menus from the North was reflected in higher production levels of this food group in the region. A strong correspondence was also noted between menus and consumption patterns. The production and consumption of legumes was higher in the North, however, this regional difference was not reflected in the menus. Green leafy vegetables were notably scarce in menus; over 70% and 30% of the menus in the North and South, respectively, did not include any items from this group. Whilst this again reflects production patterns, the overall low inclusion of items from this food group in the menus may be attributed to the very short shelf life and low weight to volume ratio.

The overall market demand for HGSF as a percentage of national production may not appear to be significant. For example, the total GSFP demand for legumes based on current menus according to the simulation results in Fig. 4 would constitute 2.83% of legume (cowpea and groundnut) production in Ghana in 2012/13 (Food Balance Sheet 2012/2013, SIRD, MoFA). However, the HGSF procurement platform and systems may strengthen supply chains and market integration for different commodities. Where appropriate HGSF procurement can help create shorter supply chains through more direct farm linkages with Farmer Based Organizations (FBOs) for certain commodities ensuring better prices for farmers and improved value for money.

Cowpea provides a useful illustration for this proposition. While the production of cowpeas (a legume) is concentrated in Northern Ghana, all menus studied included this commodity and similar quantities were used in both the North and the South. Market integration for cowpea in Ghana is known to be particularly weak with most of the price benefit accruing to intermediaries with wholesalers holding a monopoly on storage to the detriment of both producers and consumers (Rusike et al. 2013). Through the institutionalized and predictable demand presented by schools in Southern Ghana, HGSF may have implications for the cowpea supply chain. One mechanism may be through direct forward contracts on a school term or annual basis between school caterers in the South with identified Farmer Based Organizations in the cowpea producing regions in the North. Roundtable negotiations between school feeding caterers and FBOs could support the development of such contracts (Gelli et al. 2016). Complementary capacity building and technology interventions that strengthen farmer-based organization and smallholder participation may help reduce overall fixed transaction costs generally and for specific commodities (Gelli et al. 2016). It has been suggested that the aggregate supply response to induced changes in transactions costs are likely to exceed those of other policies such as trade and price (Barett 2008).

Similarly, given the high prevalence of tomato in all school menus, HGSF procurement could be used to strengthen value chains through enhanced linkages with smallholder farmers and localized procurement at the district level. Processing of tomatoes may also serve to reduce post-harvest loss and year-around usage of locally-grown tomatoes. Tomato production is reported to be highly seasonal leading to significant imports from neighbouring countries. Most of the tomato paste is also imported from the European Union and China (Awo 2012). Tomato paste or canned tomatoes were present in 88% of menus studied, often in conjunction with fresh tomatoes. Okra presents another interesting example of the potential role of HGSF in driving commodity specific value chain. It featured in more than half of menus, which is substantial given its highly seasonal availability limited to five months a year and post-harvest losses estimated to be up to 34% (Affognon et al. 2015; Kitinoja 2010).

The study also highlights several critical issues related to HGSF and other agricultural policies seeking to enhance local procurement. HGSF policies often emphasize the need to procure from smallholder farmers, but in many countries including Ghana there is no uniform policy to define and identify smallholder farmers (Singh 2011). In the absence of such definitions and reliable compliance mechanisms, a broad channelling of procurement towards smallholder farmers can be achieved through appropriate commodity selection as certain food groups, such as legumes and dark green leafy vegetables, which are more suitable to small farm production.

Finally, some major limitations of this study need to be emphasized. The small samples in terms of the menus and the number of respondents to the household survey did not allow for analysis at the district level. Instead, the analysis was undertaken at the regional level (North versus South). Data from only one point in time was available and changes over time due to the HGSF intervention could not be assessed. As the food items reported in the production module and the household food consumption module were not identical, the focus of the analysis was on food groups. In addition, analysis was conducted for four individual foods which were common in the menus and included in the list of possible survey responses for both production and consumption. Given the limitations mentioned above, an important caveat here is that these findings should not be extrapolated to other similar interventions and individual commodities without a more granular commodity-specific analysis across production and marketing. Despite these limitations, the investigation does provide a foundation for future analyses, as well as for the study of variation by community and household characteristics. In particular, this study may shed light on how HGSF menus and other similar interventions may strengthen linkages between production and dietary diversity at the local level over time, as well as reduce post-harvest losses and create incentives for on-farm storage.

## Footnotes

1 Data from the Ghana Education Service is available from the website: http://www.ghanaeducationdata.com/ (link downloaded November 28, 2015).

2 Also known as white beans in Ghana.

3 Also known as peanuts.
